# Predicting extensive metastasis in postoperative oligometastatic colorectal cancer

**DOI:** 10.1007/s00384-025-04841-w

**Published:** 2025-02-26

**Authors:** Rencai Fan, Chenkai Mao, Jiaqi Zhang, Min Dai, Rong Zhang, Xinran Wang, Jiaxin Dai, Shicheng Li, Zhixiang Zhuang

**Affiliations:** 1 Center for Cancer Diagnosis and Treatment, The Second Affiliated Hospital of Soochow University, No.1055, Sanxiang Road, Gusu District, Soochow, 215004 Jiangsu Province P.R. China; 2 Department of Respiratory Medicine, Wu Zhong People’s Hospital, No. 61 Dongwu North Road, Wu Zhong District, Soochow, 215100 Jiangsu Province P.R. China; 3 Department of Oncology, The Nuclear Industry 417 Hospital, No. 5 Kangfu Road, Lintong District, Xi’an, Shaanxi Province 710600 P.R. China

**Keywords:** Colorectal cancer, Oligometastasis, Extensive metastasis, Risk factors, Prediction model

## Abstract

**Purpose:**

Oligometastatic colorectal cancer (OMCRC) patients can achieve long-term disease control with multidisciplinary treatment. However, the development of extensive metastasis worsens prognosis and restricts treatment options. This study aims to develop a predictive model for extensive metastasis in OMCRC to assist in clinical decision-making.

**Methods:**

Clinical and pathological data for OMCRC patients were collected from the Second Affiliated Hospital of Soochow University. Patients were randomly divided into training and testing cohorts. Risk factors for extensive metastasis were identified through LASSO regression analysis and COX regression analysis. Three predictive models were developed in the training cohort and validated in the testing cohort: COX regression analysis, Extreme Gradient Boosting (XGBoost), and Survival Support Vector Machine (SurvSVM). Finally, the optimal model was visualized with the nomogram.

**Results:**

A total of 214 patients with OMCRC were enrolled in the study. Four independent risk factors were identified: whether surgery has been undertaken following oligometastasis (WST), histological type (HT), carcinoembryonic antigen at the last follow-up (CAE at last-FU), and preoperative albumin to globulin ratio (Preop-AGR). In the testing cohort, the COX model (1-year AUC = 0.82, 3-year AUC = 0.72, 5-year AUC = 0.85, mean AUC = 0.80) performed best. Decision curve analysis (DCA) confirmed the net benefit of the Cox model, and the nomogram provided accurate predictions of metastasis risk.

**Conclusion:**

CAE at last-FU, Preop-AGR, HT, and WST are independent risk factors for extensive metastasis in OMCRC. The nomogram model incorporating risk factors can assist clinicians in developing optimal treatment for OMCRC patients.

**Supplementary Information:**

The online version contains supplementary material available at 10.1007/s00384-025-04841-w.

## Introduction

CRC (colorectal cancer) has become a significant public health challenge both in China and worldwide [1, 2]. Approximately 15–30% of CRC patients have metastases, and 20–50% of initially localized CRC cases eventually develop metastases [3–5]. The liver is the most common site of metastasis for CRC, followed by the lungs, peritoneum, and distant lymph nodes. Most patients with metastatic CRC are not candidates for R0 surgical resection and instead receive systemic therapy [6], resulting in poorer prognoses [7, 8].

Numerous clinical studies have shown that patients with isolated liver metastasis from CRC can achieve long-term survival following surgery and systemic treatment [9]. Similar outcomes have been observed in other secondary sites, such as lung metastases [7, 10–12]. The 2022 ESMO Clinical Practice Guidelines recommend that physicians assess whether patients with metastatic CRC are in a state of oligometastatic disease (OMD), defined as controlled primary tumor, all metastases being safely treatable by local methods, and metastases limited to no more than two sites [7]. Similarly, the NCCN Guidelines guide clinical practice based on the resectability of metastases [13]. In the treatment of OMCRC, a broader range of local therapies, such as radiotherapy and interventional techniques, are utilized for managing metastatic lesions, rather than relying solely on surgery. A multidisciplinary treatment approach that includes systemic chemotherapy, targeted therapy, surgical resection of metastases, and local ablation can offer patients the possibility of long-term disease control and even clinical cure [4]. It is important to note that despite undergoing surgery and systemic treatment, 55 to 80% of patients with OMCRC will experience tumor recurrence and metastasis [14, 15]. For patients with persistent OMCRC, additional local and systemic treatments are still considered beneficial. However, when patients develop extensive multi-organ metastases, palliative systemic therapy is required, and the prognosis is often poorer. Therefore, identifying risk factors for the progression to extensive metastasis in patients with OMCRC and early prediction of extensive metastatic events are particularly crucial.

Through retrospective studies and clinical trials, identifying prognostic risk factors and developing predictive models have become widely applied in cancer patient management. These tools include the Breast Cancer 21-Gene Recurrence Score, the Postoperative Recurrence Risk Score for CRC Liver Metastases, and the Endoscopic Resection Curative Scoring for Early Gastric Cancer, among others [16–20]. Essentially, these tools stratify risk for specific patient subgroups, aiding in clinical follow-up, guiding patient prognosis expectations, or identifying high-risk individuals suitable for clinical trials. Extensive research indicates that clinical data such as TNM staging, tumor pathological parameters, serum tumor markers like CEA, and peripheral blood inflammatory markers are closely related to the prognosis of CRC. Prognostic models constructed from these clinical data also guide medical decision-making. However, there is relatively limited research on prognostic factors specific to OMCRC. Existing studies mainly focus on oligometastatic patients with specific organ metastases or on the risk of disease progression in oligometastatic patients. Furthermore, these studies emphasize preoperative measurements of various serum markers, while the utilization of dynamic measurement data throughout the disease progression is insufficient. This clearly overlooks the particularities of the oligometastatic state in CRC and the dynamic nature of the disease [21–27].

This study retrospectively analyzed the clinical characteristics and longitudinal serological marker data of patients with oligometastatic colorectal cancer (OMCRC) treated at the Second Affiliated Hospital of Soochow University. The objective is to elucidate the correlation between these biomarkers and the risk of extensive metastasis and to develop a robust clinical prediction model. The model can facilitate personalized treatment planning, enable the accurate identification of OMCRC patients at high risk for extensive metastasis, and guide the implementation of optimal treatment and follow-up strategies, thereby enhancing patient outcomes and quality of life.

## Materials and methods

### Study design

**Fig. 1:**
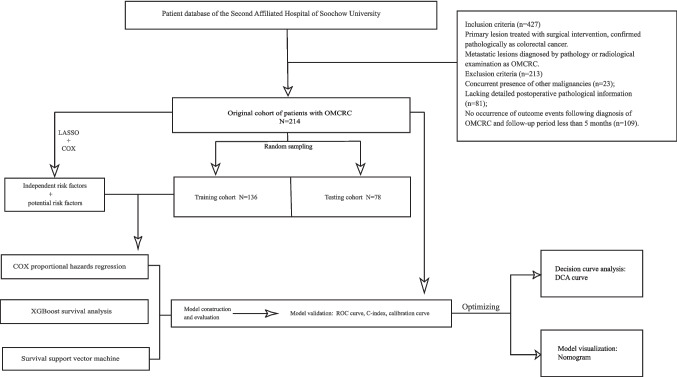
Flowchart of the study

The retrospective analysis comprehensively reviewed the medical records, hematological data, pathological information, and imaging results of 427 patients diagnosed with oligometastatic colorectal cancer at the Second Affiliated Hospital of Soochow University between January 1, 2005, and December 31, 2022. All patients were selected based on strict inclusion and exclusion criteria. Further exclusions included 23 patients with concurrent other malignancies undergoing antitumor treatment, 81 patients lacking detailed pathological information post-primary tumor surgery, and 109 patients with a follow-up period of less than 5 months. Ultimately, 214 patients met the study criteria. The detailed research process is outlined in Fig. 1. In accordance with the Equator Network guidelines, this study adheres to the Transparent Reporting of a Multivariable Prediction Model for Individual Prognosis or Diagnosis (TRIPOD) guidelines and was approved by the Medical Ethics Committee of the Second Affiliated Hospital of Soochow University (approval number [JD-HG-2024–034]). All patients provided informed consent upon admission, allowing their medical history, test results, and imaging data to be used for research purposes, with their personal information kept confidential.

### Event definitions

All patients underwent imaging examinations, including computed tomography (CT), magnetic resonance imaging (MRI), and positron emission tomography-computed tomography (PET-CT), or pathological examination of metastatic lesions, to confirm the diagnosis of OMCRC. For patients diagnosed with oligometastasis through imaging, a retrospective comparison was conducted, analyzing all imaging data, and the initial oligometastatic time was determined based on the first imaging evidence. Both synchronous and metachronous oligometastasis patients were included in the study, regardless of whether the metastatic lesions received treatment. Additionally, this study considers oligorecurrence as a special subtype of OMCRC and therefore includes patients with oligorecurrence in the analysis. Patients with no outcome events after the diagnosis of oligometastasis and a total follow-up time of less than 5 months, as well as those with other malignancies, were excluded from the study.

In this study, patients with OMCRC were defined according to the traditional criteria set forth in the 2022 ESMO Clinical Practice Guidelines: primary tumors under control, 1 to 5 metastatic sites (if the metastatic sites can be safely treated, the number can exceed 5), and all metastatic sites amenable to local treatment with no more than 2 locations involved. OMCRC patients with a disease-free survival (DFS) of 6 months or less after radical surgery were classified as having synchronous OMCRC, while those with a DFS greater than 6 months were classified as having metachronous OMCRC. In this paper, patients with OMCRC refer to those who experience tumor recurrence at the colorectal site post-surgery, with recurrence sites meeting the criteria for oligometastasis (no further distinction will be made from OMCRC patients).

In this study, an endpoint event is defined as the occurrence of extensive metastasis involving multiple sites after the diagnosis of OMCRC, including more than two metastatic sites, or two metastatic sites where the lesions are deemed inoperable upon evaluation [4]. The primary endpoint for evaluating patient outcomes is the event-free survival (EFS), which is the time from the diagnosis of oligometastasis to the occurrence of the endpoint event. Follow-up time begins from the initial pathological diagnosis of CRC and continues until the patient experiences extensive metastasis, death, or loss to follow-up. Due to the limitations of this retrospective study in distinguishing whether deaths are cancer-related and the different risks associated with cancer-related deaths and extensive metastasis, both death and loss to follow-up are considered censored events in the analysis of risk factors for extensive metastasis.

### Clinical variables enrollment

The candidate variables in this study are listed in Supplementary Table 1, with all variables based on clinical, epidemiological, or clinical research evidence. Serum marker data were collected at six time points: preoperative, postoperative, after the first postoperative treatment, at the time of oligometastasis, after oligometastasis treatment, and at the last follow-up. For patients with synchronous oligometastasis at the diagnosis of CRC, serum marker data were collected at four time points: preoperative (at the time of oligometastasis), postoperative, after the first postoperative treatment (after oligometastasis treatment), and at the last follow-up. Preoperative measurements refer to values within 4 weeks before the surgery, while postoperative measurements refer to values before the first antitumor treatment. If the patient did not receive antitumor treatment, measurements from the first follow-up within 4 weeks after surgery were used. To reduce the impact of myelosuppression following antitumor treatment, postoperative measurements were defined as values before the second antitumor treatment; if antitumor treatment was not given, measurements from the second follow-up within 4 weeks after surgery were used. Measurements at the time of metastasis refer to values within 2 weeks before and after the discovery of oligometastasis, while measurements after metastasis treatment refer to values before the second treatment after oligometastasis. Measurements at the last follow-up refer to values within 2 weeks before or after the occurrence of the outcome event; if the outcome event did not occur, the value closest to the censoring event was used. If tumor markers in the hematological data exceed the measurement limit, the measurement limit was recorded. In this study, there were 4 patients with T1 stage, 5 patients with clinical stage I, and 4 patients with well-differentiated adenocarcinoma, resulting in a small sample size and low statistical power. Therefore, T1 and T2 stages were combined into T1 + 2 stage; clinical stage I and clinical stage II were combined into clinical stage 1 + 2; moderately differentiated adenocarcinoma and well-differentiated adenocarcinoma were combined into moderately to well-differentiated adenocarcinoma.

### Data preprocessing

Serum tumor marker data in cancer patients often exhibit extreme values and substantial variability among different patients due to disease progression. Additionally, the hematological data have varying magnitudes and ranges. Therefore, in this study, logarithmic transformation was applied to the hematological data [28]. Similarly, due to the significant differences in the diameters (measured in mm) of oligometastatic lesions depending on their locations, the maximum diameter of these lesions was also logarithmically transformed. Logarithmic transformation reduces the absolute value of the data without altering its nature or relationships, enhancing the comparability of variables with different units, and improving normality and homogeneity of variance [29]. The formula for the logarithmic transformation of serum tumor markers and the maximum diameter of oligometastatic lesions in this study is *X*1 = ln(*X*); for blood cell counts, due to the presence of zero values, we used the formula *X*1 = ln(*X* + 2). Here, *X* represents the original value of the candidate variable, and *X*1 represents the value after logarithmic transformation for subsequent analysis.

To address missing data under the assumption of missing at random, we used the Markov Chain Monte Carlo (MCMC) simulation method for multiple imputation [30–32]. Variables with more than 40% missing data were excluded from the study, resulting in a final inclusion of 71 variables (Supplementary Table 2). The imputation model incorporated all other candidate variables and outcome measures. For continuous data, we employed the predictive mean matching (PMM) method, iterating five times. For categorical data, we used the random forest (RF) method, also iterating five times. Post-imputation, we performed intergroup comparisons (Supplementary Table 2), finding no statistically significant differences between any of the candidate variables (two-sided *P*-value ≥ 0.05). Subsequent data analyses were conducted using these imputed data, and the platelet-lymphocyte ratio (PLR), lymphocyte-monocyte ratio (LMR), lymphocyte-albumin product (LA), albumin-globulin ratio (AGR), C-reactive protein-albumin ratio (CRA).

### Selection of risk factors

The study employed a combination of LASSO and Cox proportional hazards regression analysis to investigate the risk factors for extensive metastasis following oligometastasis [33]. The outcome was a binary variable, analyzed as a continuous variable in the regression analysis, with EFS treated as a time variable in Cox proportional hazards regression. The analysis included age, sex, reason for medical consultation (RMC), Clinical stage, TNM stage, tumor sidedness (TS), total number of detected lymph nodes (TNDLN), number of positive lymph nodes (NPLN), the number of cancerous nodules (NCN), HT, whether the tumor exhibits vascular invasion (WVI), whether the tumor exhibits neural invasion (WNI), whether the pathology indicates mucinous adenocarcinoma (WPMA), whether postoperative treatment is regular (WPTIS), postoperative treatment plan (PTP), OE, number of oligometastatic lesions (NOL), maximum diameter of oligometastatic lesions (MDOL), WST, and time-series features (CEA, CA50, CA125, CA199, RBC, PLR, LMR, LA, AGR, and CAR). Categorical variables were included in the regression analysis as dummy variables.

The occurrence of extensive metastasis metastasis was used as the dependent variable, with EFS as the time variable. All candidate variables were included in the LASSO regression model and subjected to tenfold cross-validation. The optimal variables for the LASSO model were determined by examining Log(lambda) and partial likelihood deviance, and these variables were selected for further study [34].

Univariate Cox proportional hazards regression analysis was conducted on the candidate variables selected by LASSO regression to explore their association with EFS. Hazard ratios (HR) and *p*-values were used as evaluation metrics. Variables with clinical or statistical significance from univariate analysis (HR > 1.3 or < 0.7, or *p*-value < 0.05) were considered potential risk factors for EFS. Subsequently, multivariate Cox proportional hazards regression analysis was performed to adjust for potential risk factors and estimate adjusted *p*-values and HRs. Variables with a p-value < 0.05 and HR > 1.1 or HR < 0.9 were considered independent risk factors for EFS. Finally, Kaplan–Meier curves (KM curves) were used to validate the predictive value of each risk factor [35]. The Log-rank test was employed to compare survival curves between groups for significant differences [36].

### Model development

A training cohort was created using a random sampling method, consisting of 136 patients. The unselected samples formed the testing cohort (*n* = 78), while the original dataset (ALL cohort) served as the second testing cohort. All identified risk factors were included in the training cohort for model construction. Dummy variables were created for categorical variables. Three model construction methods were employed: Cox proportional hazards regression, eXtreme Gradient Boosting (XGBoost) survival analysis, and Survival Support Vector Machine (SurvSVM). Cox regression is a non-parametric method commonly used in survival analysis, suitable for various data types and distributions [37]. The XGBoost algorithm minimizes a loss function to learn weak classifiers and then combines them into a strong classifier to improve accuracy and generalization [38]. SVM is a supervised learning algorithm that finds the optimal hyperplane by maximizing the margin, thus classifying and performing regression analysis on data points [39]. The XGBoost survival analysis model and SurvSVM model integrate machine learning classification and regression capabilities with survival analysis methods, effectively handling right-censored survival data [40–42].

#### Development of the Cox model

Using a multivariate Cox proportional hazards regression approach, with extensive metastasis as the dependent variable and EFS as the time variable, all risk factors were incorporated into the model development in the training cohort. Based on the risk coefficients and clinical parameters, linear predictions (Prognosis Index, PI) were calculated for each patient in the training, testing, and ALL cohorts, and this value was used as the Cox Regression Risk Score (COXRiskScore, CRS). The median CRS for each cohort was used as the cutoff point to classify patients into high-risk and low-risk groups across the three cohorts.

#### Development of the XGBoost model

For the XGBoost model, hyperparameter tuning was initially performed to select parameters including the number of iterations (nrounds), feature column ratio (colsample_bytree), minimum child weight (min_child_weight), learning rate (eta), minimum loss reduction required for a split (gamma), sample ratio (subsample), and maximum depth of the tree (max_depth). The tuning process was evaluated using tenfold cross-validation to ensure the model’s robustness and generalizability. After determining the optimal hyperparameter configuration, the XGBoost model was fitted on the training cohort, with early stopping rounds (early_stopping_rounds) set to prevent overfitting and enhance model efficiency. The gain for each split node was calculated to assess the contribution of the selected features, and a variable importance plot was created to visually display the significance of each risk factor in the XGBoost model. Similarly, risk scores (XGBoostRiskScore, XGBRS) for the XGBoost model were calculated for patients in the training, testing, and ALL cohorts. The median XGBRS for each cohort was used as the cutoff point to classify patients into high-risk and low-risk groups across the three cohorts.

#### Development of the SurvSVM model

A SurvSVM model for survival analysis was developed using regression methods to predict EFS and outcome status, a technique referred to as SVCR in VanBelle et al.’s literature [42]. The model regularization parameter was set to 1, with an additive kernel function selected, and the quadprog solver was used to solve the quadratic optimization problem derived from the model’s support vector formulation to determine the optimal classification hyperplane or regression function. The SurvSVM model incorporated all risk factors and was fitted on the training cohort. The predicted risk ranks for patients in the training, testing, and ALL cohorts were calculated. In the SurvSVM model, a higher predicted risk rank corresponds to a longer survival time, so the predicted risk ranks were used as the SurvSVM Risk Score (SVMRS) in subsequent analyses. The SVMRS was calculated for the training, testing, and ALL cohorts. The median SVMRS for each cohort was used as the cutoff point to classify patients into high-risk and low-risk groups across the three cohorts.

### Testing, evaluation, and visualization of predictive models

Model validation and evaluation were conducted in the training cohort, testing cohort, and ALL cohort. The models’ discriminative ability was assessed using the C-index and the AUC curve to evaluate their ability to distinguish between different risk groups [43]. Calibration curves were used to assess the models’ calibration, ensuring the consistency between predicted probabilities and observed outcomes [44]. KM curves were plotted to evaluate the models’ event prediction and risk stratification capabilities across different datasets. After evaluating the overall performance of the three models across the three cohorts, the optimal model was selected for DCA. The DCA curves were used to assess the net benefit of clinical decisions based on the model [45], and a nomogram was employed to visually represent the best model [46].

### Statistical analysis

All statistical analyses in this study were performed using R version 4.3.1, employing packages such as “glmnet,” “survival,” “xgboost,” “survivalsvm,” and “rms” [47–53]. Continuous data following a normal distribution were described as mean ± standard deviation, and comparisons between two groups were made using the Student’s *t*-test. For continuous data not following a normal distribution, data were described as median (interquartile range), and the Wilcoxon rank-sum test was used for comparisons between two groups. Categorical data were described as frequency (percentage) and analyzed using the chi-square test for comparisons between two groups. If the expected frequency of categorical data was too small to meet the chi-square test conditions, Fisher’s exact test was used. A two-sided *p*-value of < 0.05 was considered statistically significant unless otherwise specified.

## Results

### The characteristics of patients

Table 1 presents the basic characteristics of the patients and their distribution in the training, testing, and ALL cohort. The proportion of patients with extensive metastasis is higher in the training and ALL cohorts compared to the testing cohort. In the testing cohort, the number of patients with extensive metastasis is equal to the number of patients without. The median EFS is similar across the three cohorts, with the training cohort having an EFS of 25 months and both the testing and ALL cohort having an EFS of 26 months. Most patients in the three cohorts are in clinical stage IV and T stage IV, with moderately to well-differentiated adenocarcinoma being the predominant HT. The proportion of male patients is higher than that of female patients. Further inter-cohort comparisons revealed no statistically significant differences between the cohorts (Table 1, *p* ≥ 0.05).

**Table 1:** Clinical information in training, testing, and ALL cohort

	Training cohort	Testing cohort	ALL cohort	Methods for inter-group comparison	*P*-value
Total	136	78	214		
Outcome				Chisq. (2 *df*) = 0.39	0.824
Non-extensive metastasis	62 (45.6)	39 (50)	101 (47.2)		
Extensive metastasis	74 (54.4)	39 (50)	113 (52.8)		
EFS				Kruskal–Wallis test	0.955
Median (IQR)	25 (14, 38.2)	26 (15, 39)	26 (14.2, 39)		
Gender				Chisq. (2 *df*) = 0.61	0.736
Male	78 (57.4)	49 (62.8)	127 (59.3)		
Female	58 (42.6)	29 (37.2)	87 (40.7)		
Clinical_stage				Chisq. (4 *df*) = 1.09	0.895
1 + 2	28 (20.6)	12 (15.4)	40 (18.7)		
3	38 (27.9)	21 (26.9)	59 (27.6)		
4	70 (51.5)	45 (57.7)	115 (53.7)		
T_stage				Chisq. (4 *df*) = 3.47	0.482
1 + 2	10 (7.4)	12 (15.4)	22 (10.3)		
3	56 (41.2)	29 (37.2)	85 (39.7)		
4	70 (51.5)	37 (47.4)	107 (50)		
OE				Chisq. (6 *df*) = 3.59	0.732
Intestinal metastasis	15 (11)	3 (3.8)	18 (8.4)		
Hepatic metastasis	84 (61.8)	53 (67.9)	137 (64)		
Pulmonary metastasis	32 (23.5)	18 (23.1)	50 (23.4)		
Metastasis of other organs	5 (3.7)	4 (5.1)	9 (4.2)		
WST				Chisq. (2 *df*) = 0	0.999
Non-surgical treatment	78 (57.4)	45 (57.7)	123 (57.5)		
Surgical treatment	58 (42.6)	33 (42.3)	91 (42.5)		
NCN				Chisq. (2 *df*) = 5.25	0.073
No cancer nodules detected	94 (69.1)	65 (83.3)	159 (74.3)		
Detected cancer nodules	42 (30.9)	13 (16.7)	55 (25.7)		
HT				Chisq. (2 *df*) = 0.28	0.868
Poorly differentiated adenocarcinoma	19 (14)	13 (16.7)	32 (15)		
Moderately to well-differentiated adenocarcinoma	117 (86)	65 (83.3)	182 (85)		
NPLN				Kruskal–Wallis test	0.122
Median (IQR)	1 (0, 2)	1 (0, 3.8)	1 (0, 3)		
Preop_PLR				Kruskal–Wallis test	0.527
Median (IQR)	4.4 (4, 4.9)	4.3 (3.9, 4.9)	4.4 (4, 4.9)		
Preop_LA				Kruskal–Wallis test	0.750
Median (IQR)	4.6 (4, 5.2)	4.8 (4.2, 5.4)	4.7 (4.1, 5.3)		
Preop_AGR				Kruskal–Wallis test	0.973
Median (IQR)	1.1 (1, 1.2)	1.1 (1.1, 1.2)	1.1 (1, 1.2)		
Postop_CEA				Kruskal–Wallis test	1.000
Median (IQR)	1.6 (1.1,2.7)	1.5 (1.1,2.6)	1.6 (1.1,2.7)		
CEA_after_1st_Postop_Tx				Kruskal–Wallis test	0.360
Median (IQR)	1.6 (1.3, 3.1)	1.8 (1.4, 3.7)	1.7 (1.3, 3.2)		
CA199_after_1st_Postop_Tx				Kruskal–Wallis test	0.864
Median (IQR)	3 (2.2, 4)	2.9 (2.4, 4.1)	3 (2.2, 4)		
CEA_at_Oligo				Kruskal–Wallis test	0.881
Median (IQR)	2.2 (1.6, 3.9)	2.2 (1.7, 3.5)	2.2 (1.6, 3.6)		
CEA_after_Oligo_Tx				Kruskal–Wallis test	0.840
Median (IQR)	1.7 (1.4, 2.8)	1.8 (1.3, 2.5)	1.7 (1.4, 2.6)		
CA50_at_LFU				Kruskal–Wallis test	0.980
Median (IQR)	3.1 (1.8,5)	3.3 (2.1, 4.6)	3.2 (1.9, 4.6)		
CEA_at_LFU				Kruskal–Wallis test	1.000
Median (IQR)	2.5 (1.6, 4.5)	2.4 (1.7, 4.4)	2.5 (1.6, 4.5)		

*IQR *interquartile range

### Selection of risk factors

#### LASSO regression analysis

**Fig. 2:**
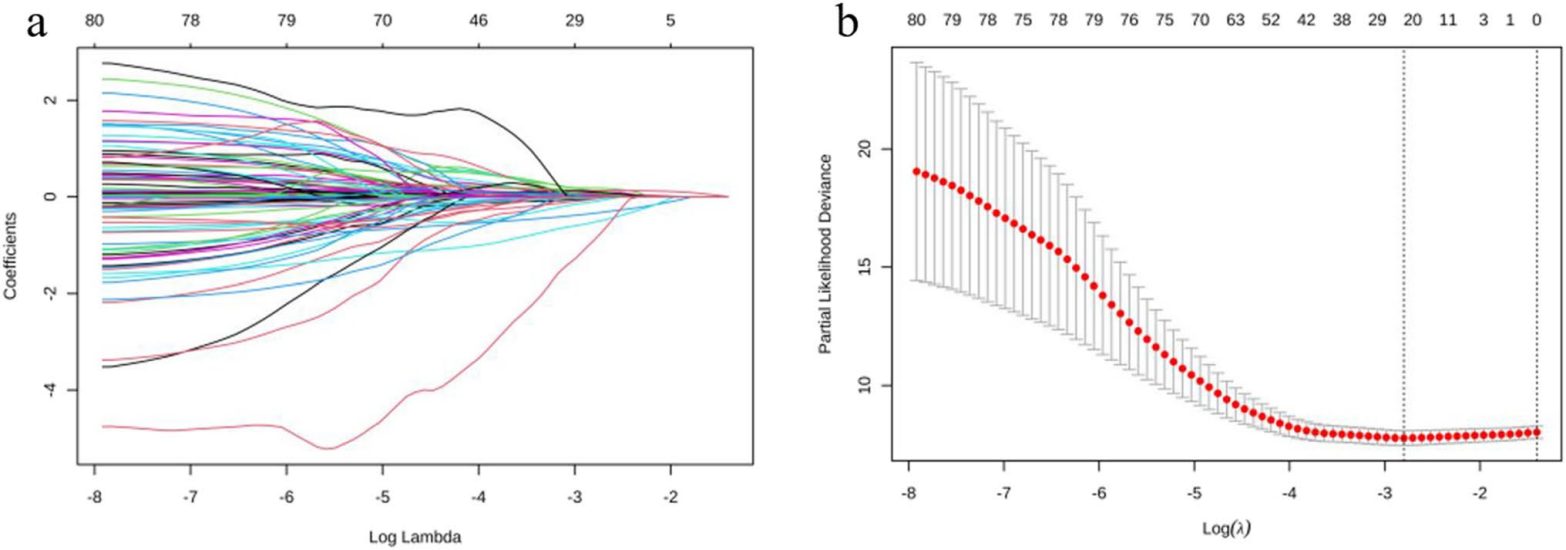
LASSO regression analysis and cross-validation (*80 parameters were included in the LASSO regression due to dummy variable settings)

A total of 71 candidate variables were included in this study, and the LASSO regression model was fitted using data from all patients (Fig. 2a). Through tenfold cross-validation, the minimum lambda value (lambda min) was 0.061, corresponding to 20 non-zero coefficients; the minimum lambda minus standard error (lambda min-se) was 0.246, corresponding to 0 non-zero coefficients (Fig. 2b). A total of 18 candidate variables were identified for subsequent analysis, including gender, clinical stage, T stage, OE, WST, NCN, HT, NPLN, preoperative CEA (Postop-CEA), CEA after the first postoperative treatment (CEA after 1st Postop-Tx), CA199 after the first postoperative treatment (CA199 after 1st Postop-Tx), CEA at the time of oligometastasis (CEA at Oligo), CEA after oligometastasis treatment (CEA after Oligo-Tx), CA50 at last follow-up (CA50 at LFU), CEA at LFU, preoperative PLR (Preop-PLR), preoperative LA (Preop-LA), and Preop-AGR.

#### COX proportional hazards regression analysis

**Fig. 3:**
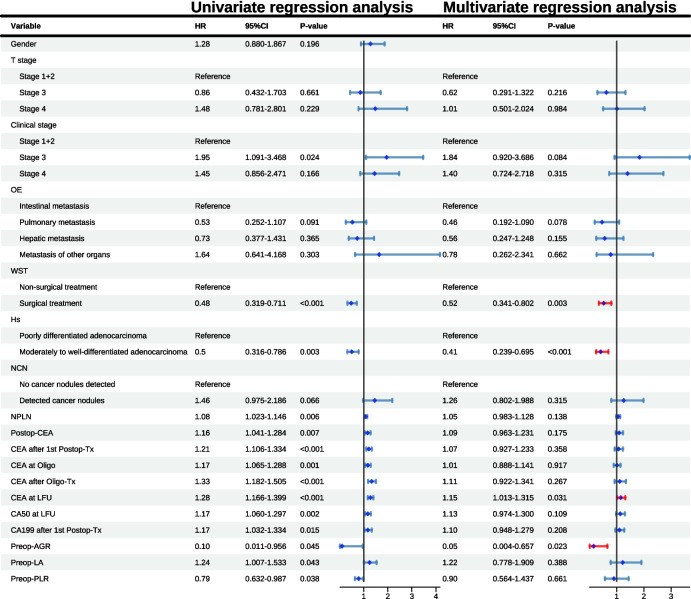
Forest plot of univariate and multivariate Cox proportional hazards regression analyses

Univariate Cox proportional hazards regression analysis was performed on 18 candidate variables. Based on the selection criteria (*P* < 0.05 or HR > 1.3 or HR < 0.7), 17 of the 18 candidate variables, excluding gender, were identified as potential risk factors for EFS (Fig. 3). Subsequently, these 17 potential risk factors were included in a multivariate Cox proportional hazards regression analysis. The proportional hazards assumption test (Supplementary Table 3) and the Schoenfeld residual plot (Supplementary Fig. 1) indicated that all variables met the proportional hazards assumption (*P* > 0.05), and the global significance test confirmed good model fit (GLOBAL *P* > 0.05). The multivariate Cox proportional hazards regression analysis (Fig. 3) identified 4 independent risk factors associated with EFS (*P* < 0.05 and HR > 1.1 or HR < 0.9): WST (surgical treatment vs non-surgical treatment: HR = 0.52, *P* = 0.003); HT (moderately to well-differentiated adenocarcinoma vs poorly differentiated adenocarcinoma: HR = 0.41, *P* < 0.001); CEA at LFU (HR = 1.15, *P* = 0.031); and Preop-AGR (HR = 0.05, *P* = 0.023). The global significance test of the multivariate Cox proportional hazards regression analysis had a *P* value of < 0.001, indicating that these 4 independent risk factors have predictive value for patients’ EFS.

#### Validation of risk factors

**Fig. 4:**
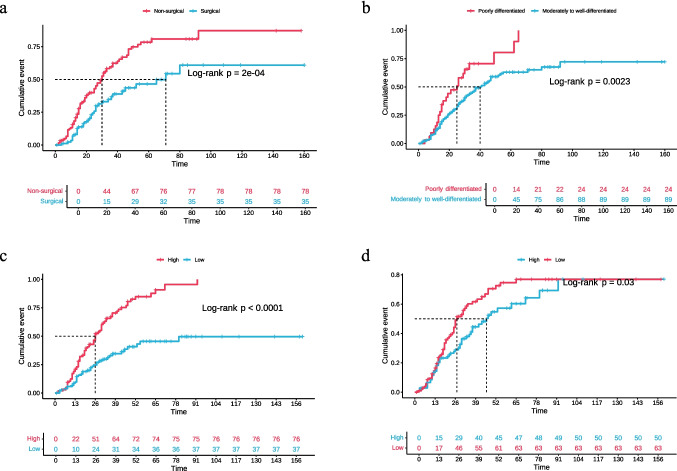
** a** KM curve of patients grouped by WST; **b** KM curve of patients grouped by HT; **c** KM curve of patients grouped by median CEA at LFU; **d** KM curve of patients grouped by median Preop-AGR

KM curves were plotted for the four independent risk factors (Fig. 4a–d) and the thirteen potential risk factors (Supplementary Fig. 2a-m) in relation to EFS, stratified by the median if the risk factor was continuous. The KM curves for WST, HT, and CEA at LFU showed clear separation throughout, with statistically significant differences (*P* < 0.05), indicating strong independent risk stratification capability. Despite crossing at 0–13 months and after 91 months, the KM curve for Preop-AGR showed overall good separation with statistical significance, also demonstrating good independent risk stratification capability (*P* < 0.05). Among the 13 potential risk factors, CEA after 1st Postop-Tx, CEA at Oligo, CEA after Oligo-Tx, and CA50 at LFU also showed some risk stratification capability. Although the curves for T stage and OE showed statistically significant differences (*P* < 0.05) and trends consistent with Cox proportional hazards regression analysis, there was crossing within the variable groups. The KM curves for NCN, Postop-CEA, and CA199 after 1st Postop-Tx showed good separation but lacked statistical significance (*P* > 0.05), suggesting limited risk stratification capability. The remaining potential risk factors (clinical stage, NPLN, Preop-PLR, Preop-LA) showed poor separation, with no statistically significant differences, indicating poor independent risk stratification capability.

### Model evaluation

#### Assessment and comparison of discrimination

**Fig. 5:**
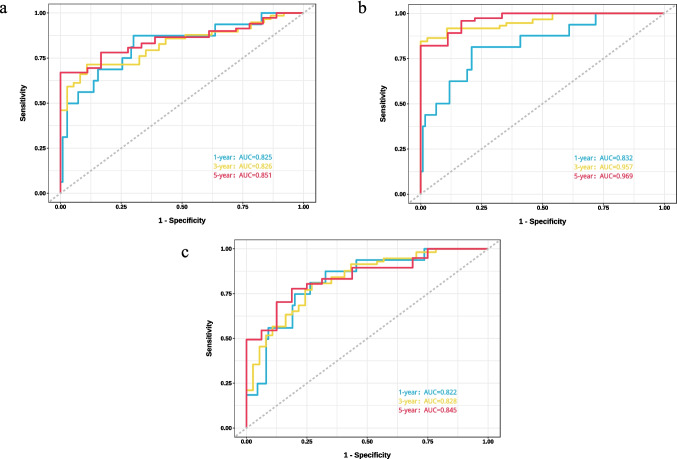
** a** ROC curves for the COX model at 1, 3, and 5 years in the training cohort; **b** ROC curves for the XGBoost model at 1, 3, and 5 years in the training cohort; **c** ROC curves for the SurvSVM model at 1, 3, and 5 years in the training cohort

All three models achieved an AUC greater than 0.8 in the training cohort, indicating good discriminatory power (Fig. 5a–c). Among them, the XGBoost model performed best in the training set (1-year AUC = 0.83, 3-year AUC = 0.96, 5-year AUC = 0.97, mean AUC = 0.92). The COX model (1-year AUC = 0.83, 3-year AUC = 0.83, 5-year AUC = 0.85, mean AUC = 0.84) and the SurvSVM model (1-year AUC = 0.82, 3-year AUC = 0.83, 5-year AUC = 0.85, mean AUC = 0.83) showed similar performance. The mean AUC of the three prediction models in the testing cohort and the ALL cohort were both greater than 0.7 (Supplementary Fig. 3a-f), indicating good discriminatory power in the ALL cohort and the unfamiliar testing cohort. This consistent performance demonstrates the models’ robustness and generalizability, verifying their reliability in real-world scenarios. In the testing cohort, the COX model (1-year AUC = 0.82, 3-year AUC = 0.72, 5-year AUC = 0.85, mean AUC = 0.80) performed best. Although the SurvSVM model had a 3-year AUC < 0.70 in the testing cohort (1-year AUC = 0.76, 3-year AUC = 0.63, 5-year AUC = 0.87, mean AUC = 0.75), its mean AUC was still better than the XGBoost model (1-year AUC = 0.75, 3-year AUC = 0.73, 5-year AUC = 0.70, mean AUC = 0.73). In the ALL cohort, the SurvSVM model (1-year AUC = 0.80, 3-year AUC = 0.76, 5-year AUC = 0.87, mean AUC = 0.81) and the COX model (1-year AUC = 0.82, 3-year AUC = 0.79, 5-year AUC = 0.86, mean AUC = 0.82) had similar performance, with the XGBoost model performing best (1-year AUC = 0.81, 3-year AUC = 0.88, 5-year AUC = 0.89, mean AUC = 0.86).

The COX model exhibited a C-index greater than 0.7 in the training, testing, and ALL cohorts, consistent with the ROC curve evaluations (Table 2, training cohort C-index = 0.756, testing cohort C-index = 0.724, ALL cohort C-index = 0.744, mean C-index = 0.741). Both the SurvSVM and XGBoost models had C-index values below 0.7 in the testing cohort, but the SurvSVM model (Table 2, training cohort C-index = 0.747, testing cohort C-index = 0.670, ALL cohort C-index = 0.719, mean C-index = 0.712) demonstrated superior overall performance across the three datasets compared to the XGBoost model (Table 2, training cohort C-index = 0.731, testing cohort C-index = 0.612, ALL cohort C-index = 0.688, mean C-index = 0.677).

**Table 2:** C-index of three models in training, testing, and ALL cohort

	COX model	XGBoost model	SurvSVM model
Training cohort	0.756	0.731	0.747
Testing cohort	0.724	0.612	0.670
ALL cohort	0.744	0.688	0.719
Mean	0.741	0.677	0.712

#### Assessment and comparison of calibration

**Fig. 6:**
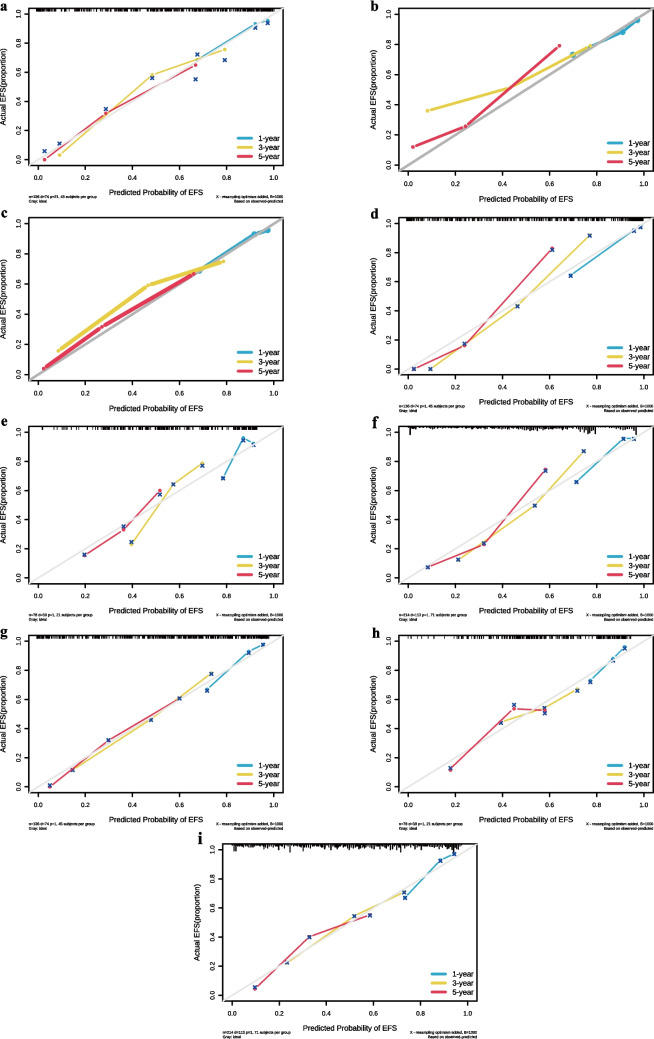
** a** Calibration curves at 1, 3, and 5 years for the COX model in the training cohort; **b** calibration curves at 1, 3, and 5 years for the COX model in the testing cohort; **c** calibration curves at 1, 3, and 5 years for the COX model in the ALL cohort; **d** calibration curves at 1, 3, and 5 years for the XGBoost model in the training cohort; **e** calibration curves at 1, 3, and 5 years for the XGBoost model in the testing cohort; **f** calibration curves at 1, 3, and 5 years for the XGBoost model in the ALL cohort; **g** calibration curves at 1, 3, and 5 years for the SurvSVM model in the training cohort; **h** calibration curves at 1, 3, and 5 years for the SurvSVM model in the testing cohort; **i** calibration curves at 1, 3, and 5 years for the SurvSVM model in the ALL cohort

In the training cohort, the 1-year and 5-year calibration curves of the COX model closely aligned with the 45-degree line, indicating good agreement between observed and predicted risks across the entire range of predicted risks (Fig. 6a). However, the model showed some miscalibration in the 3-year calibration curve; it slightly understimated risk when the predicted risk was between 0.3 and 0.7 and slightly overestimated risk when the predicted risk was between 0.1 and 0.2 (Fig. 6a). In the testing cohort, the model showed slight miscalibration of observed risk at 1 year (Fig. 6b). The 3-year and 5-year calibration curves in the testing cohort suggested slight underestimation of risk by the model (Fig. 6b). In the ALL cohort, the model slightly underestimated the observed risk at 3 years (Fig. 6c). The 1-year and 5-year calibration curves for the entire cohort indicate good model calibration (Fig. 6c).

In the training cohort, the 1-year calibration curve of the XGBoost model showed some overestimation of risk (Fig. 6d). Additionally, the model’s 3-year and 5-year calibration curves in the training cohort performed poorly. At the 3-year mark, the model underestimated risk for high-risk patients (predicted risk > 0.5) and overestimated risk for low-risk patients (predicted risk < 0.5). By the 5-year mark, the model underrestimated risk for patients with predicted risk > 0.35 and overestimated risk for those with predicted risk < 0.35 (Fig. 6d). The XGBoost model exhibited similar patterns in the ALL cohort, with poor 3-year and 5-year calibration curves (Fig. 6f). In the testing cohort, the model performed poorly across most regions for 1-year, 3-year, and 5-year predicted risks (Fig. 6e), showing mixed overestimation and underestimation of risks. These results indicate that the XGBoost survival analysis model has poor calibration, especially when predicting unfamiliar data, and its performance deteriorates over time.

The SurvSVM model’s 1-year calibration curve in the training cohort closely aligned with the 45-degree line, indicating good agreement between observed and predicted risks across the entire range of predicted risks (Fig. 6g). The 3-year and 5-year calibration curves in the training cohort showed slight miscalibration. At 3 years, the model slightly underestimated risk when the predicted risk was > 0.6 and slightly overestimated risk when the predicted risk was < 0.6 (Fig. 6g). In the ALL cohort, the SurvSVM model’s 3-year calibration curve showed good alignment between observed and predicted risks across the entire range (Fig. 6i). However, the 1-year and 5-year calibration curves exhibited mixed overestimation and underestimation of risks. In the testing cohort, the SurvSVM model showed poor calibration across most areas for 1-year, 3-year, and 5-year predicted risks, with mixed overestimation and underestimation of risks across the entire risk spectrum (Fig. 6h).

The KM curves for the COX model, XGBoost model, and SurvSVM model in the training cohort, testing cohort, and ALL cohort all showed significant separation, with corresponding *P-*values indicating statistical significance (Supplementary Fig. 4a-i, *P* < 0.001). This suggests that all three models have good predictive and discriminatory power, effectively distinguishing between individuals with different risk levels across various cohorts.

### Decision curve analysis

**Fig. 7:**
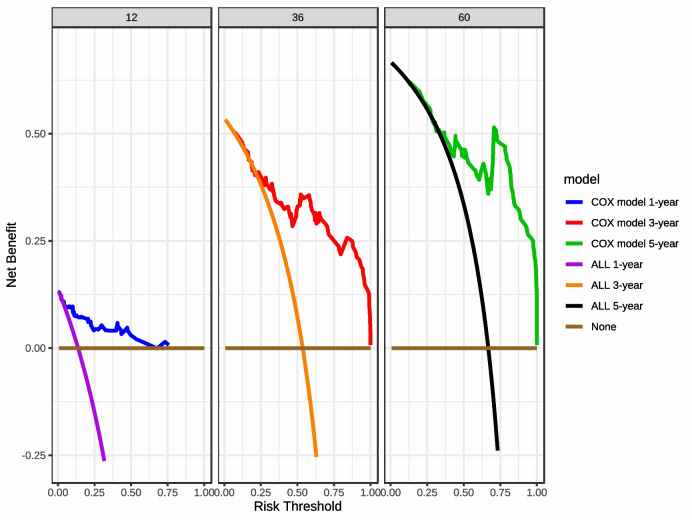
1-year, 3-year, and 5-year DCA curves for the COX model

The COX model maintained consistent discriminatory power while also demonstrating a certain level of calibration across all cohorts. Therefore, it was selected as the optimal model for decision curve analysis, and a DCA curve was plotted (Fig. 7). The 1-year and 5-year DCA curves for the COX model are above the horizontal line None and the diagonal line ALL. The 3-year DCA curve intersects with the diagonal line ALL when the threshold is between 0.125 and 0.25, but at other threshold values, the 3-year DCA curve remains above the horizontal line None and the diagonal line ALL. Overall, although the net benefit of clinical decision-making based on the model predictions decreases with increasing thresholds, there is almost always a net benefit across the full range of thresholds when making clinical decisions based on the COX model’s predictions.

### Model visualization

**Fig. 8:**
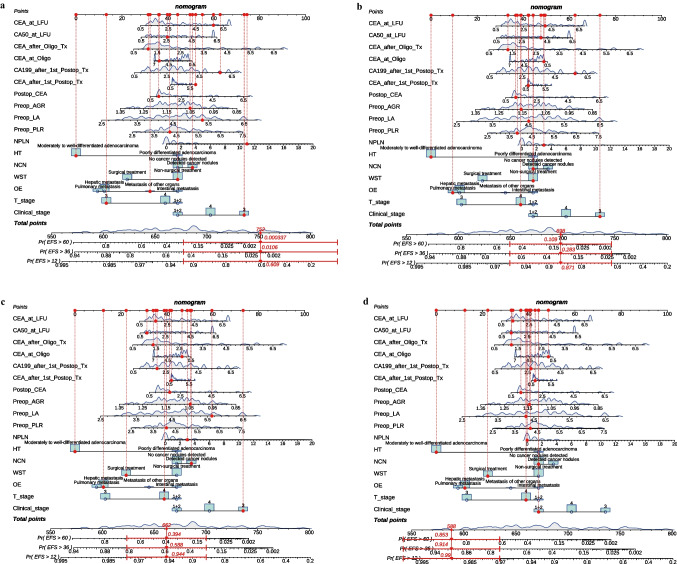
** a** 1-year, 3-year, and 5-year EFS for patient A; **b** 1-year, 3-year, and 5-year EFS for patient B; **c** 1-year, 3-year, and 5-year EFS for patient C; **d** 1-year, 3-year, and 5-year EFS for patient D

In the training cohort, nomograms were constructed using the COX method. Based on patients’ EFS and outcome status, four patients from the testing cohort were selected in natural order for the nomogram display. Patient A had an EFS of 3 months and developed extensive metastases; patient B had an EFS of 26 months and developed extensive metastases; patient C had an EFS of 43 months and developed extensive metastases; patient D had an EFS of 72 months and was censored. The total score for patient A’s nomogram was 752, predicting a 1-year EFS probability of 0.609 and probabilities of less than 0.01 for 3-year and 5-year EFS (Fig. 8a). The total score for patient B’s nomogram was 698, predicting a 1-year EFS probability of 0.871, a 3-year probability of 0.283, and a 5-year probability of 0.109 (Fig. 8b). The total score for patient C’s nomogram was 662, predicting a 1-year EFS probability of 0.944, a 3-year probability of 0.588, and a 5-year probability of 0.394 (Fig. 8c). The total score for patient D’s nomogram was 588, predicting a 1-year EFS probability of 0.99, a 3-year probability of 0.914, and a 5-year probability of 0.853 (Fig. 8d). This demonstrates that even in the unfamiliar testing cohort, the nomogram based on the COX model can quickly and accurately predict patient outcomes at various risk levels.

## Discussion

CRC is a globally prevalent cancer with a high incidence and mortality rate. Approximately half of CRC patients experienced tumor metastasis during their lifetime, with the liver and lungs being the most common sites of metastasis [54, 55]. OMCRC is a specific subtype of CRC where local treatment of metastatic lesions combined with systemic therapy can significantly improve patient prognosis. As the disease progresses, patients with OMCRC may still develop extensive metastases, severely impacting prognosis [8]. Thus, there is an urgent need for personalized treatment and follow-up plans for patients with OMCRC. The study conducted a retrospective analysis of survival data and clinical information from patients with OMCRC to identify clinical risk factors leading to extensive metastasis and developed three clinical prediction models. Although all models demonstrated good discriminatory ability, comprehensive performance evaluation revealed that in low-latitude clinical settings, COX proportional hazards regression analysis may offer good discrimination and reasonable calibration.

Research on OMCRC is still in its infancy. In the study, clinical risk factors associated with CRC recurrence and metastasis were selected based on clinical guidelines and literatures as candidates. Gender and age were included as basic patient information, as previous studies have highlighted their predictive value for CRC metastasis [56, 57]. In terms of tumor pathology data, TNM staging, clinical stage, HT, NCN, NCN, and WVI or WNI have been widely used in various tumor metastasis risk prediction models [58–60]. Additionally, tumor metastasis sites, synchronous metastasis, and subsequent treatment plans are also associated with local recurrence of CRC [61–64]. Furthermore, this study included various tumor markers such as CEA and CA199. CEA is frequently considered in CRC research, with elevated levels linked to increased incidence of CRC [65]. Studies have shown that elevated Preop-CEA levels are associated with higher rates of CRC recurrence and liver metastasis [66, 67]. CA199 has also been confirmed to have prognostic value for gastrointestinal tumors [68]. Inflammation, particularly chronic inflammation, is considered a significant risk factor for cancer development. Inflammatory markers such as PLR, LMR, PNI, and CRP are closely related to CRC prognosis [69–72]. However, existing research often relies on static data from specific time points and overlooks the dynamic nature of tumor progression. Time series analysis has gained attention from researchers, with some studies making progress in analyzing dynamic tumor evolution through time series data [73–76]. Therefore, to more accurately predict the risk of extensive metastasis in OMCRC, we included hematological data from several key time points during patient follow-up as candidate factors. Subsequent analysis confirmed that the time series behavior of CEA has predictive value for the risk of extensive metastasis in OMCRC.

This study employed LASSO regression analysis for feature selection, minimizing the impact of multicollinearity on subsequent regression analyses [34]. Variables selected through LASSO regression underwent univariate COX proportional hazards regression analysis to examine their relationship with EFS. Statistical indicators and clinical relevance were used as screening criteria to assess the association between risk factors and EFS, a method widely applied in medical research [77, 78]. Ultimately, multivariate COX proportional hazards regression analysis identified 4 independent risk factors and 13 potential risk factors for extensive metastasis in patients with OMCRC. The four independent risk factors provided distinct predictive value for the risk of extensive metastasis, while the potential risk factors offered supplementary value when combined with the independent risk factors in assessing the risk of extensive metastasis. Tumor HT has been found to correlate closely with patient prognosis in various cancers [79]. In CRC, poorly differentiated adenocarcinoma is associated with an increased risk of liver metastasis, and patients with poorly differentiated adenocarcinoma often have a lower overall survival rate compared to those with well-differentiated adenocarcinoma [80]. Similarly, this study found that patients with poorly differentiated adenocarcinoma had a higher risk of extensive metastasis compared to those with moderately to well-differentiated adenocarcinoma (Fig. 3, moderately to well-differentiated adenocarcinoma vs poorly differentiated adenocarcinoma: HR = 0.41, *P* < 0.001). CEA is one of the most commonly used tumor markers for assessing CRC, closely related to patient prognosis. Previous research has identified Preop-CEA levels greater than 200 as a significant predictor of CRC recurrence risk [81]. Although this study did not find Preop-CEA to be predictive of the risk of extensive metastasis in OMCRC, Postop-CEA (Fig. 3, HR = 1.09, *P* = 0.175), CEA after 1st Postop-Tx (Fig. 3, HR = 1.07, *P* = 0.358), CEA at Oligo (Fig. 3, HR = 1.01, *P* = 0.917), and CEA after Oligo-Tx (Fig. 3, HR = 1.11, *P* = 0.267) demonstrated potential value in assessing the risk of extensive metastasis. Furthermore, elevated CEA during follow-up in patients with OMCRC was associated with an increased risk of extensive metastasis (Fig. 3, HR = 1.15, *P* = 0.031). AGR, a combination of nutritional and inflammatory markers, is commonly used to assess immune nutrition and inflammatory status. Previous studies have found AGR to be closely related to prognosis in various cancers, including CRC, gastric, and breast cancers [82–85]. Consistent with previous research, this study found that high AGR status before curative surgery for CRC was associated with a lower risk of extensive metastasis after oligometastasis (Fig. 3, HR = 0.05, *P* = 0.023). Curative surgery remains the preferred treatment for CRC patients. For those with resectable liver metastases, surgical resection of metastatic lesions remains the only potential curative option [86]. Retrospective studies on metastatic CRC patients undergoing multiple lines of treatment suggest that surgery for metastatic lesions is an important prognostic factor [87]. Similarly, this study found that surgery for metastatic lesions was associated with a reduced risk of extensive metastasis in patients with OMCRC (Fig. 3, HR = 0.52, *P* = 0.003).

The study has certain limitations. (1) While the study suggests that factors such as WST, HT, CEA at LFU, and Preop-AGR are associated with the risk of extensive metastasis in patients with OMCRC, it cannot establish causal relationships between these risk factors and the risk of extensive metastasis. (2) In this study, we did not consider genomic data. Genomic data is crucial for understanding the occurrence, progression, and treatment response of cancer [88–90]. In CRC research, genomic data can provide key insights into the molecular characteristics of tumors, mutations, and drug sensitivity. (3) The study was single-center, and results from a single-center study may not be generalizable to other populations, healthcare settings, or geographic regions.

Future research should incorporate genomic data to gain a more comprehensive understanding of the molecular characteristics of tumors, mutations, and drug sensitivity. Moreover, since this study was conducted at a single center, its findings may not be fully applicable to other populations, healthcare settings, or geographic regions. Therefore, multi-center studies involving diverse populations are essential to validate these results and improve the robustness and generalizability of the predictive model across various clinical environments.

## Conclusions

In the study, WST, HT, CAE at last-FU, and Pre-AGR were identified as independent risk factors of extensive metastasis in OMCRC. The COX model can predict extensive metastasis with good discrimination and calibration. The model’s nomogram enables dynamic and accurate prediction of the risk of extensive metastasis, assisting clinicians in providing optimal personalized treatment.

## Supplementary Information

Below is the link to the electronic supplementary material.

ESM 1 (PDF 1.49 MB)

ESM 2 (PDF 203 KB)

## Data Availability

No datasets were generated or analysed during the current study.
