# Supplementary material for: Predicting extensive metastasis in postoperative oligometastatic colorectal cancer
Source: Int J Colorectal Dis. 2025 Feb 26;40(1):53. doi: 10.1007/s00384-025-04841-w (PMC11861249; doi:10.1007/s00384-025-04841-w)
Supplement: Supplementary file 1 — (PDF 1.49 MB) [file 384_2025_4841_MOESM1_ESM.pdf]

Supplementary material

Supplementary Table 1: Candidate Risk Factors

Variable	Data type	Annotation
Clinical stage<br>T stage<br>N stage<br>M stage	Categorical data	Reference the American Joint Committee on Cancer (AJCC) eighth edition TNM staging system
Whether postoperative treatment is regular (WPTIS)	Categorical data	Patients are categorized into two groups based on whether they adhere to the standard treatment protocol and whether treatment interruptions occur due to treatment reactions, economic status, or other reasons during the treatment process: regular treatment and irregular treatment
Postoperative treatment plan (PTP)	Categorical data	Divided into: postoperative follow-up, postoperative adjuvant chemotherapy (including Postoperative adjuvant chemotherapy, Neoadjuvant therapy)
Oligometastasis events (OE)	Categorical data	Divided into: intestinal metastasis, hepatic metastasis, pulmonary metastasis, metastasis of other organs (including ovarian, bone, cranial, etc.)
Whether surgery has been undertaken following oligometastasis (WST)	Categorical data	Divided into: surgical treatment after oligometastasis, non-surgical treatment after oligometastasis
Reason for medical consultation (RMC)	Categorical data	Divided into: incidental findings during physical examination, abdominal pain, alterations in bowel movements (constipation, diarrhea, changes in stool consistency and frequency), and bloody stool
Tumor sidedness (TS)	Categorical data	Divided into right-sided and left-sided based on the hepatic and splenic regions
The number of cancerous nodules (NCN)	Categorical data	Divided into: detected cancer nodules, no cancer nodules detected
Whether the pathology indicates mucinous adenocarcinoma (WPMA)	Categorical data	Divided into: mucinous adenocarcinoma, non-mucinous adenocarcinoma
Histological type (HT)	Categorical data	According to the WHO histological classification of tumors (2019 edition), it is divided into: poorly differentiated adenocarcinoma , Moderately to well-differentiated adenocarcinoma (including moderately differentiated adenocarcinoma and well-differentiated adenocarcinoma)
Whether the tumor exhibits vascular invasion [illegible]	Categorical data	According to postoperative pathology, it is divided into: vascular invasion, no vascular invasion detected
Whether the tumor exhibits neural invasion	Categorical data	According to postoperative pathology, it is divided into: neural invasion, no neural invasion detected
Number of oligometastatic lesions	Categorical data	According to whether the metastatic lesions are more than one, it is divided into single lesions and multiple lesions

Timing of metastasis (TM)	Categorical data	Metastases are classified into synchronous and metachronous metastases. Synchronous metastasis is defined as the detection of metastatic tumors at the time of primary tumor diagnosis or within six months thereafter
Maximum diameter of oligometastatic lesions (MDOL)	Continuous data	Measure via imaging system and select the maximum diameter of the largest metastatic lesion. Units are in mm
Age	Continuous data	Biological age at the diagnosis of colorectal cancer
Total number of detected lymph nodes （TNDLN） Number of positive lymph nodes （NPLN）	Count data	Based on postoperative pathological data obtained, processed as continuous data
Cancer antigen 50 (CA50)	Continuous data	Data were collected at six time points: preoperative, postoperative, after the first postoperative treatment, at the time of oligometastasis, after oligometastasis treatment, and at last follow-up
Cancer antigen 125 (CA125)	Continuous data	Data were collected at six time points: preoperative, postoperative, after the first postoperative treatment, at the time of oligometastasis, after oligometastasis treatment, and at last follow-up
Carcinoembryonic antigen (CEA)	Continuous data	Data were collected at six time points: preoperative, postoperative, after the first postoperative treatment, at the time of oligometastasis, after oligometastasis treatment, and at last follow-up
Cancer antigen 199 (CA199)	Continuous data	Data were collected at six time points: preoperative, postoperative, after the first postoperative treatment, at the time of oligometastasis, after oligometastasis treatment, and at last follow-up
Red blood cell count (RBC)	Count data	Data were collected at six time points: preoperative, postoperative, after the first postoperative treatment, at the time of oligometastasis, after oligometastasis treatment, and at last follow-up
Platelet to lymphocyte ratio (PLR)	Continuous data	Platelet count/lymphocyte count (PLT/LY). Data were collected at six time points: preoperative, postoperative, after the first postoperative treatment, at the time of oligometastasis, after oligometastasis treatment, and at last follow-up
Lymphocyte to monocyte ratio (LMR)	Continuous data	Lymphocyte count/monocyte count (LY/MC). Data were collected at six time points: preoperative, postoperative, after the first postoperative treatment, at the time of oligometastasis, after oligometastasis treatment, and at last follow-up
Lymphocyte to albumin product (LA)	Continuous data	Lymphocyte count * serum albumin (LY*Alb). Data were collected at six time points: preoperative, postoperative, after the first postoperative treatment, at the time of oligometastasis, after oligometastasis treatment, and at last follow-up
Albumin to globulin ratio (AGR)	Continuous data	Serum albumin/serum globulin (Alb/Glob). Data were collected at six time points: preoperative, postoperative, after the first postoperative treatment, at the time of oligometastasis, after oligometastasis treatment, and at last follow-up
C-reactive protein to serum albumin ratio (CAR)	Continuous data	C-reactive protein/serum albumin (CRP/Alb). Data were collected at six time points: preoperative, postoperative, after the first postoperative treatment, at the time of oligometastasis, after oligometastasis treatment, and at last follow-up

Supplementary Table 2: omparison of Data Before and After Imputation

	Imputed data	Original data	Methods for inter-group comparison	P-value
Total	214	214		
Age			Ranksum test	1
median(IQR)	62 (55,69)	62 (55,69)		
Gender			Chisq. (1 df) = 0	0.97
Male	127 (59.3)	126 (59.2)		
Female	87 (40.7)	87 (40.8)		
Clinical stage			Chisq. (3 df) = 0.01	1
1	5 (2.3)	5 (2.4)		
2	35 (16.4)	34 (16)		
3	59 (27.6)	58 (27.4)		
4	115 (53.7)	115 (54.2)		
T-stage			Fisher's exact test	1
1	5 (2.3)	4 (1.9)		
2	17 (7.9)	16 (7.8)		
3	85 (39.7)	83 (40.3)		
4	107 (50)	103 (50)		
N-stage			Chisq. (2 df) = 0.04	0.98
0	74 (34.6)	72 (34.8)		
1	103 (48.1)	98 (47.3)		
2	37 (17.3)	37 (17.9)		
M-stage			Chisq. (1 df) = 0	1
0	99 (46.3)	99 (46.3)		
1	115 (53.7)	115 (53.7)		
WPTIS			Chisq. (1 df) = 0.18	0.67
Irregular treatment	25 (11.7)	25 (13.1)		
Regular treatment	189 (88.3)	166 (86.9)		
PTP			Chisq. (2 df) = 0.01	0.10
Postoperative follow-up	22 (10.3)	22 (10.3)		
Postoperative adjuvant chemotherapy	154 (72)	154 (72.3)		
Neoadjuvant therapy	38 (17.8)	37 (17.4)		

	Imputed data	Original data	Methods for inter-group comparison	P-value
OE			Chisq. (3 df) = 0	1
Intestinal metastasis	18 (8.4)	18 (8.4)		
Hepatic metastasis	137 (64)	137 (64)		
Pulmonary metastasis	50 (23.4)	50 (23.4)		
Metastasis of other organs	9 (4.2)	9 (4.2)		
WST			Chisq. (1 df) = 0	1
Non-surgical treatment	123 (57.5)	123 (57.5)		
Surgical treatment	91 (42.5)	91 (42.5)		
RMC			Chisq. (3 df) = 0.24	0.97
Incidental findings during physical examination	25 (11.7)	24 (12.1)		
Abdominal pain	51 (23.8)	49 (24.7)		
Alterations in bowel movements	53 (24.8)	45 (22.7)		
Bloody stool	85 (39.7)	80 (40.4)		
TS			Chisq. (1 df) = 0	0.97
Right-sided	70 (32.7)	70 (32.9)		
Left-sided	144 (67.3)	143 (67.1)		
NCN			Chisq. (1 df) = 0.09	0.76
No cancer nodules detected	159 (74.3)	146 (73)		
Detected cancer nodules	55 (25.7)	54 (27)		
WPMA			Chisq. (1 df) = 0.01	0.91
Non-mucinous adenocarcinoma	193 (90.2)	186 (89.9)		
Mucinous adenocarcinoma	21 (9.8)	21 (10.1)		
HT			Fisher's exact test	0.97
Poorly differentiated adenocarcinoma	32 (15)	32 (15.8)		
Moderately differentiated adenocarcinoma	178 (83.2)	166 (82.2)		
Well-differentiated adenocarcinoma	4 (1.9)	4 (2)		
WVI			Chisq. (1 df) = 0	0.99
No vascular invasion detected	147 (68.7)	138 (68.7)		
vascular invasion	67 (31.3)	63 (31.3)		
WNI			Chisq. (1 df) = 0.01	0.91
No neural invasion detected	166 (77.6)	155 (77.1)		
Neural invasion	48 (22.4)	46 (22.9)		

	Imputed data	Original data	Methods for inter-group comparison	P-value
NOL			Chisq. (1 df) = 0	0.99
Single lesions	70 (32.7)	66 (32.7)		
Multiple lesions	144 (67.3)	136 (67.3)		
DFS			Ranksum test	1
median(IQR)	0 (0,16)	0 (0,16)		
EFS			Ranksum test	1
median(IQR)	26 (14.2,39)	26 (14.2,39)		
TNDLN			Ranksum test	0.68
median(IQR)	13 (9,18)	13 (9,18)		
NPLN			Ranksum test	0.97
median(IQR)	1 (0,3)	1 (0,2)		
Preop-CEA			Ranksum test	0.51
median(IQR)	2.1 (1.5,3.2)	2.2 (1.7,2.9)		
Preop-CA199			Ranksum test	0.73
median(IQR)	2.9 (1.9,4.4)	2.9 (2.4,3.6)		
Preop-Glob			Ranksum test	0.05
median(IQR)	3.4 (3.3,3.5)	3.3 (3.2,3.4)		
Preop-Alb			Ranksum test	0.709
median(IQR)	3.8 (3.7,3.8)	3.8 (3.7,3.8)		
Preop-RBC			Ranksum test	0.11
median(IQR)	1.9 (1.8,1.9)	1.8 (1.8,1.9)		
Preop-PLT			Ranksum test	0.49
median(IQR)	5.4 (5.2,5.8)	5.4 (5.2,5.6)		

	Imputed data	Original data	Methods for inter-group comparison	P-value
Preop-LY			Ranksum test	0.45
median(IQR)	1.3 (1.1,1.4)	1.2 (1.1,1.4)		
Preop-MC			Ranksum test	0.44
median(IQR)	0.9 (0.8,1)	0.9 (0.8,0.9)		
Preop-CRP			Ranksum test	0.66
median(IQR)	2.1 (1.9,2.7)	2.1 (2,2.2)		
Postop-CEA			Ranksum test	0.52
median(IQR)	1.6 (1.1,2.7)	1.6 (1.3,2.2)		
Postop-CA199			Ranksum test	0.44
median(IQR)	2.8 (2.2,4.1)	2.7 (2.3,3.4)		
Postop-Glob			Ranksum test	0.87
median(IQR)	3.4 (3.2,3.6)	3.4 (3.3,3.5)		
Postop-Alb			Ranksum test	0.61
median(IQR)	3.8 (3.7,3.9)	3.8 (3.7,3.8)		
Postop-RBC			Ranksum test	0.19
median(IQR)	1.8 (1.8,1.9)	1.8 (1.8,1.9)		
Postop-PLT			Ranksum test	0.57
median(IQR)	5.3 (5.1,5.6)	5.3 (5.1,5.6)		
Postop-LY			Ranksum test	0.77
median(IQR)	1.2 (1.1,1.4)	1.2 (1.1,1.3)		

	Imputed data	Original data	Methods for inter-group comparison	P-value
Postop-MC			Ranksum test	0.07
median(IQR)	0.9 (0.8,0.9)	0.9 (0.8,0.9)		
Postop-CRP			Ranksum test	0.36
median(IQR)	2.1 (2,3)	2.1 (2,2.3)		
CEA after 1st Postop-Tx			Ranksum test	0.67
median(IQR)	1.7 (1.3,3.2)	1.7 (1.4,2.3)		
CA199 after 1st Postop-Tx			Ranksum test	0.29
median(IQR)	3 (2.2,4)	2.8 (2.3,3.4)		
Alb after 1st Postop-Tx			Ranksum test	0.57
median(IQR)	3.8 (3.7,3.9)	3.8 (3.7,3.9)		
RBC after 1st Postop-Tx			Ranksum test	0.69
median(IQR)	1.8 (1.7,1.9)	1.8 (1.8,1.9)		
PLT after 1st Postop-Tx			Ranksum test	0.30
median(IQR)	5.1 (4.9,5.4)	5.2 (5,5.4)		
LY after 1st Postop-Tx			Ranksum test	0.85
median(IQR)	1.2 (1.1,1.4)	1.2 (1.1,1.3)		
MC after 1st Postop-Tx			Ranksum test	0.81
median(IQR)	0.9 (0.8,0.9)	0.9 (0.8,0.9)		
MDOL			Ranksum test	0.81
median(IQR)	3 (2.5,3.7)	3 (2.5,3.6)		
CEA at Oligo			Ranksum test	0.69

	Imputed data	Original data	Methods for inter-group comparison	P-value
CEA at Oligo			Ranksum test	0.69
median(IQR)	1.9 (1.5,3.1)	1.9 (1.5,2.7)		
CA199 at Oligo			Ranksum test	0.38
median(IQR)	3 (2.3,4.2)	2.9 (2.4,3.5)		
Glob at Oligo			Ranksum test	0.44
median(IQR)	3.4 (3.3,3.6)	3.4 (3.3,3.5)		
Alb at Oligo			Ranksum test	0.71
median(IQR)	3.8 (3.7,3.9)	3.8 (3.8,3.9)		
RBC at Oligo			Ranksum test	0.30
median(IQR)	1.8 (1.8,1.9)	1.9 (1.8,1.9)		
PLT at Oligo			Ranksum test	0.40
median(IQR)	5.2 (4.9,5.4)	5.2 (4.9,5.4)		
LY at Oligo			Ranksum test	0.70
median(IQR)	1.2 (1.1,1.4)	1.2 (1.1,1.4)		
MC at Oligo			Ranksum test	0.65
median(IQR)	0.9 (0.8,0.9)	0.9 (0.8,0.9)		
CRP at Oligo			Ranksum test	0.74
median(IQR)	2 (2,2.2)	2 (2,2.1)		
CEA after Oligo-Tx			Ranksum test	0.37
median(IQR)	1.7 (1.3,2.4)	1.7 (1.4,2.5)		
CA199 after Oligo-Tx			Ranksum test	0.45
median(IQR)	2.7 (2.2,3.3)	2.8 (2.3,3.3)		

	Imputed data	Original data	Methods for inter-group comparison	P-value
Glob after Oligo-Tx			Ranksum test	0.26
median(IQR)	3.4 (3.3,3.5)	3.4 (3.3,3.5)		
Alb after Oligo-Tx			Ranksum test	0.36
median(IQR)	3.8 (3.7,3.9)	3.8 (3.7,3.9)		
RBC after Oligo-Tx			Ranksum test	0.61
median(IQR)	1.8 (1.8,1.9)	1.8 (1.8,1.9)		
PLT after Oligo-Tx			Ranksum test	0.82
median(IQR)	5.2 (4.9,5.4)	5.2 (4.9,5.4)		
LY after Oligo-Tx			t-test (198 df) = 0.02	0.99
mean(SD)	1.2 (0.2)	1.2 (0.2)		
MC after Oligo-Tx			Ranksum test	0.43
median(IQR)	0.9 (0.8,0.9)	0.9 (0.8,0.9)		
CRP after Oligo-Tx			Ranksum test	0.93
median(IQR)	2 (2,2.2)	2.1 (2,2.2)		
CA50 at LFU			Ranksum test	0.49
median(IQR)	3.2 (1.9,4.6)	3 (2.1,4)		
CA125 at LFU			Ranksum test	0.78
median(IQR)	2.7 (2.3,3.6)	2.7 (2.4,3.3)		
CEA at LFU			Ranksum test	0.10
median(IQR)	2.5 (1.6,4.5)	2.5 (1.6,4.5)		
CA199 at LFU			Ranksum test	0.89
median(IQR)	3.3 (2.5,4.5)	3.3 (2.5,4.6)		

	Imputed data	Original data	Methods for inter-group comparison	P-value
Glob at LFU			Ranksum test	0.88
median(IQR)	3.4 (3.3,3.5)	3.4 (3.3,3.5)		
Alb at LFU			Ranksum test	0.65
median(IQR)	3.8 (3.7,3.9)	3.8 (3.7,3.9)		
RBC at LFU			Ranksum test	0.82
median(IQR)	1.8 (1.7,1.9)	1.8 (1.7,1.9)		
PLT at LFU			Ranksum test	0.97
median(IQR)	5.2 (5,5.4)	5.2 (5,5.4)		
LY at LFU			Ranksum test	0.91
median(IQR)	1.2 (1,1.3)	1.2 (1,1.3)		
MC at LFU			Ranksum test	0.86
median(IQR)	0.9 (0.8,0.9)	0.9 (0.8,0.9)		
CRP at LFU			Ranksum test	0.93
median(IQR)	2.1 (2,3)	2.1 (2,3)		

IQR:Interquartile range

Supplementary Table 3 Proportional Hazards Assumption Test

Variable	P-value
Clinical stage	0.07
OE	0.11
WST	0.81
NCN	0.79
HT	0.12
NPLN	0.47
Preop-PLR	0.86
Preop-LA	0.66
Preop-AGR	0.27
Postop-CEA	0.11
CEA after 1st Postop-Tx	0.18
CA199 after 1st Postop-Tx	0.33
CEA at Oligo	0.61
CEA after Oligo-Tx	0.62
CA50 at LFU	0.61

Variable	P-value
CEA at LFU	0.06
GLOBAL	0.12

7

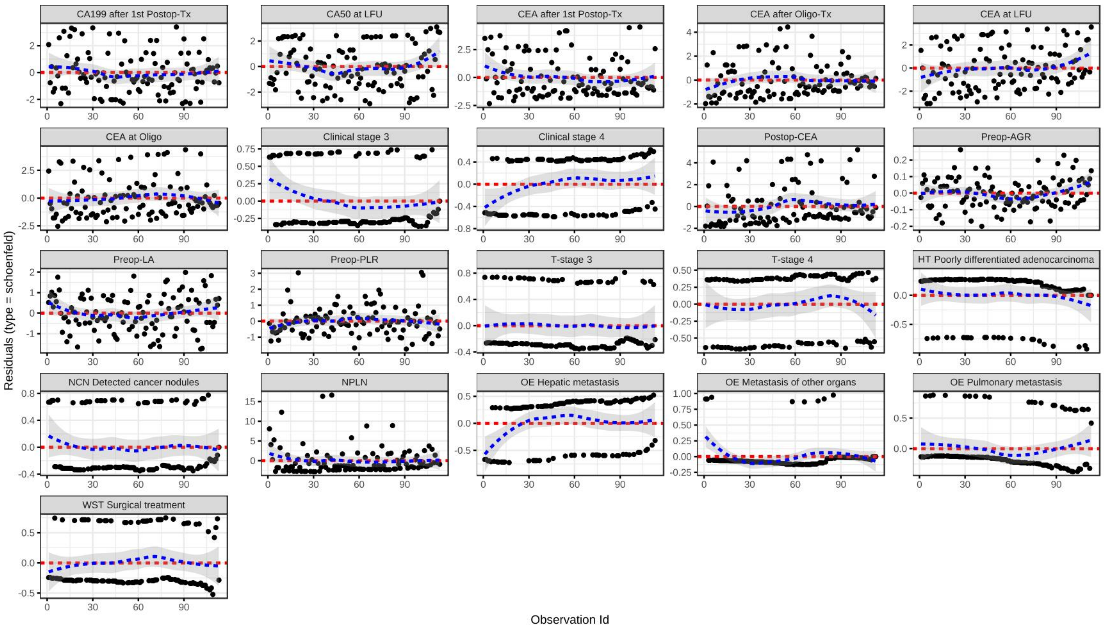

Supplementary Figure 1 Schoenfeld Residual Plot for Proportional Hazards Assumption Testing

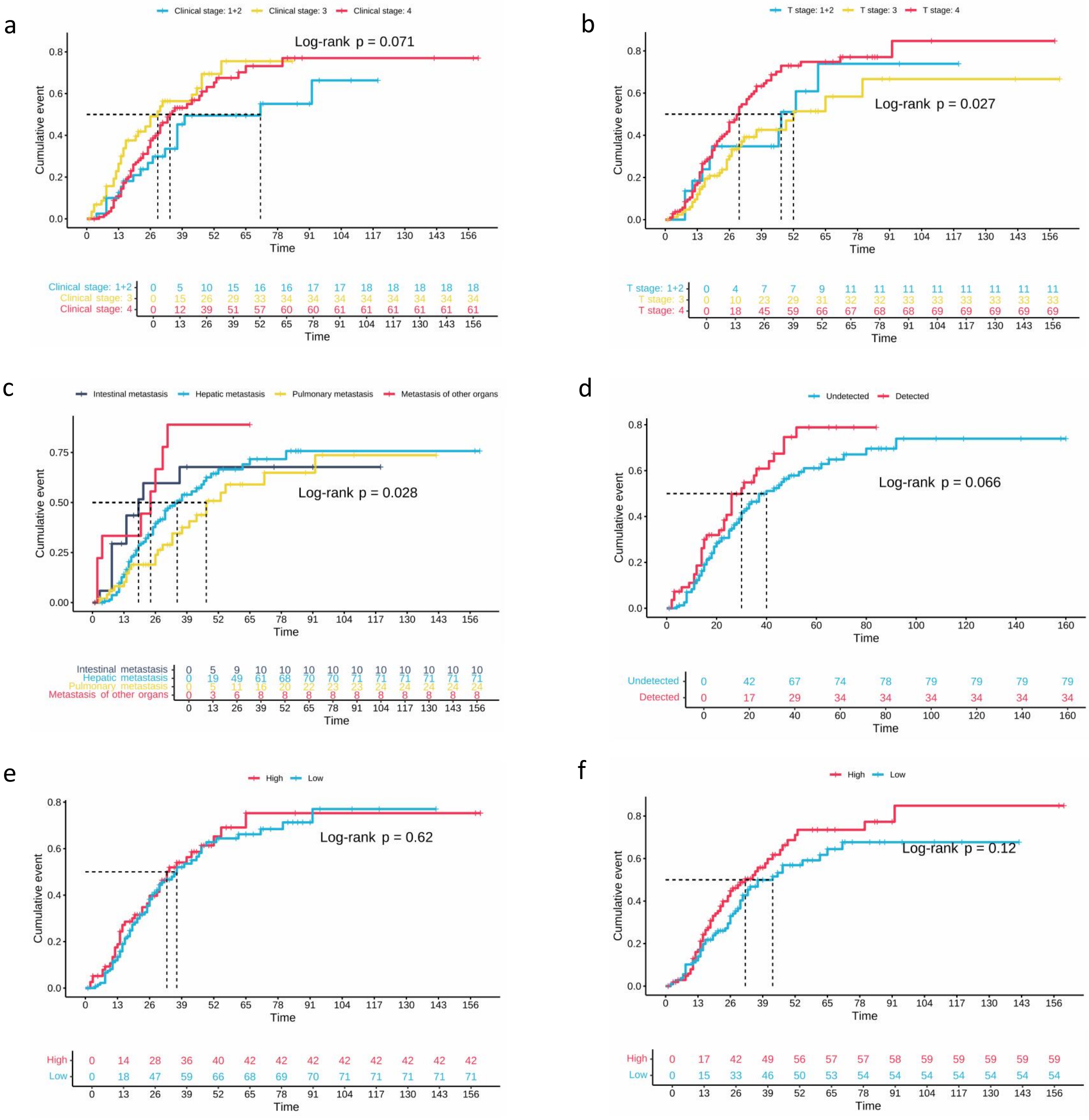

g

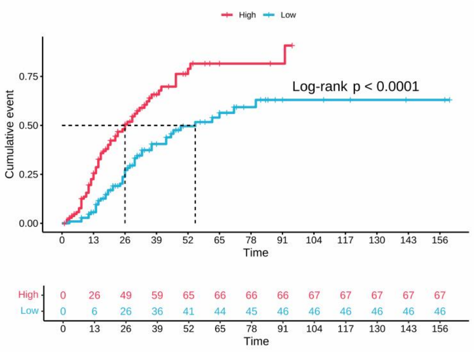

h

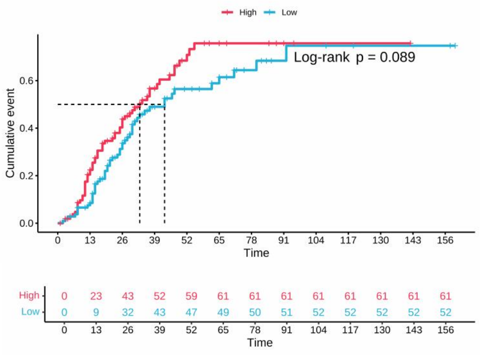

i

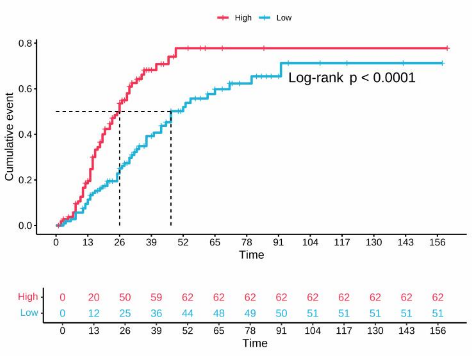

j

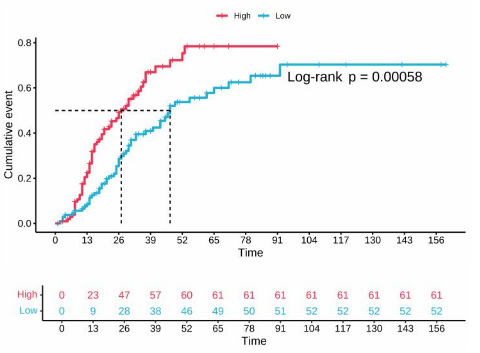

k

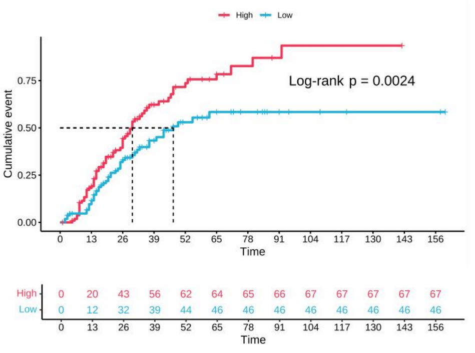

l

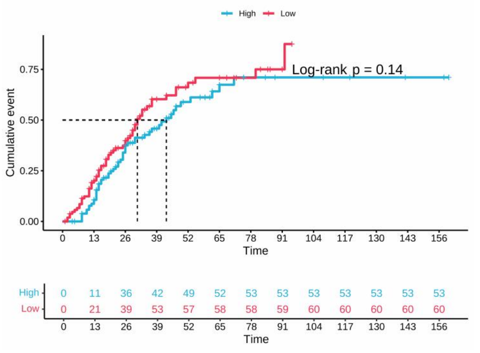

m

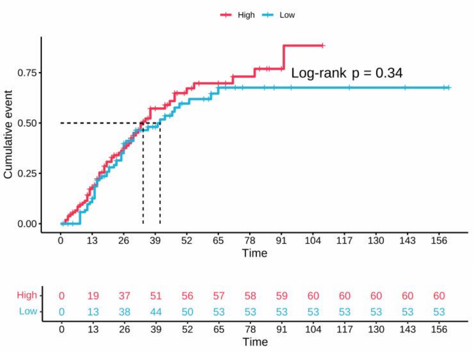

Supplementary Figure 2: (a) KM curve for patients grouped by clinical stage; (b) KM curve for patients grouped by T stage; (c) KM curve for patients grouped by OE; (d) KM curve for patients grouped by NCN; (e) KM curve for patients grouped by NPLN; (f) KM curve for patients grouped by median Postop-CEA; (g) KM curve for patients grouped by median CEA after 1st Postop-Tx; (h) KM curve for patients grouped by median CA199 after 1st Postop-Tx; (i) KM curve for patients grouped by median CEA at Oligo; (j) KM curve for patients grouped by median CEA after Oligo-Tx; (k) KM curve for patients grouped by median CA50 at LFU; (l) KM curve for

patients grouped by median Preop-PLR; (m) KM curve for patients grouped by median Preop-LA.

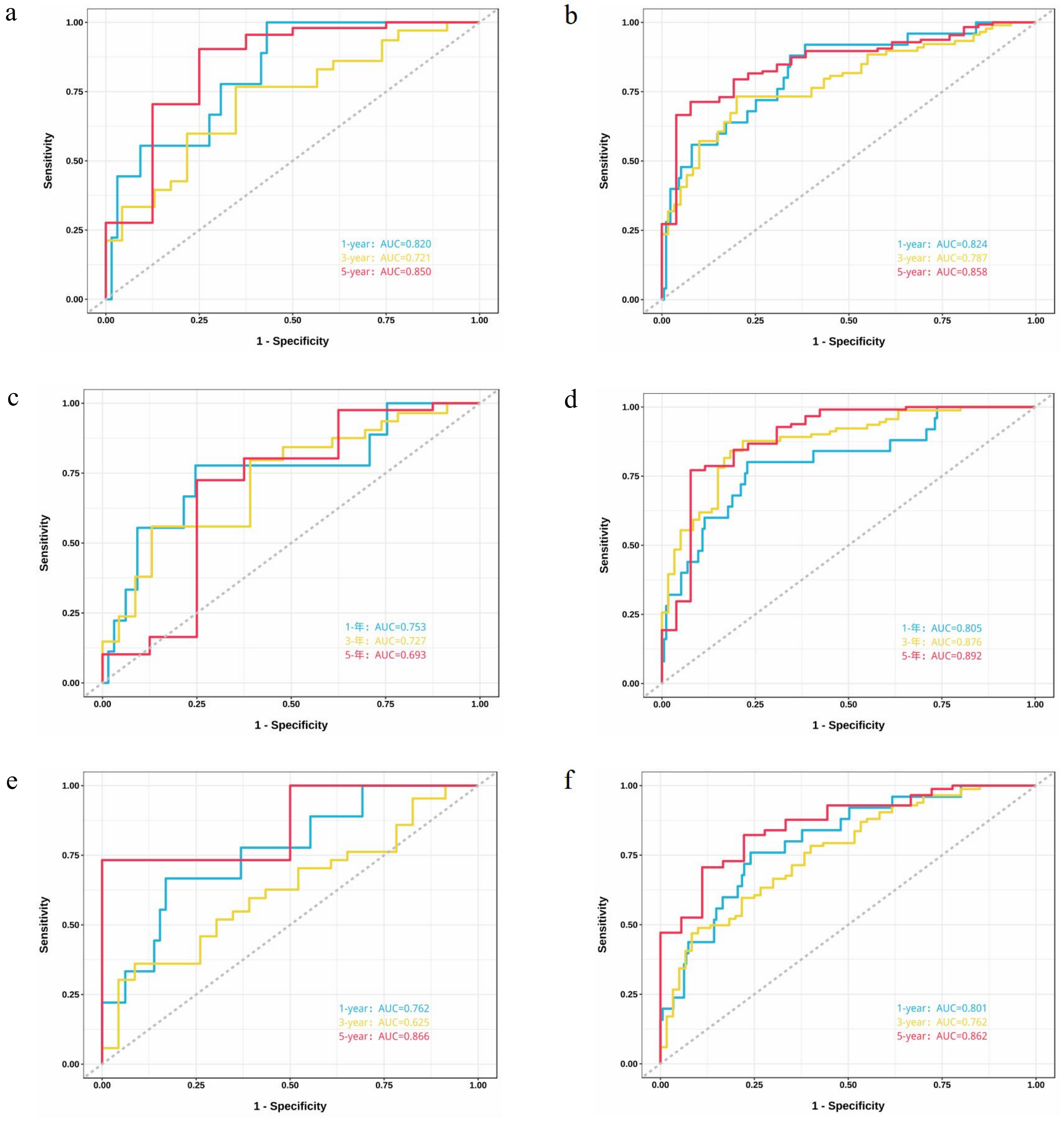

Supplementary Figure 3: (a) ROC curves for the COX model at 1, 3, and 5 years in the testing cohort; (b) ROC curves for the COX model at 1, 3, and 5 years in the ALL cohort; (c) ROC curves for the XGBoost model at 1, 3, and 5 years in the testing cohort; (d) ROC curves for the XGBoost model at 1, 3, and 5 years in the ALL cohort; (e) ROC curves for the SurvSVM model at 1, 3, and 5 years in the testing cohort; (f) ROC curves for the SurvSVM model at 1, 3, and 5 years in the ALL cohort.

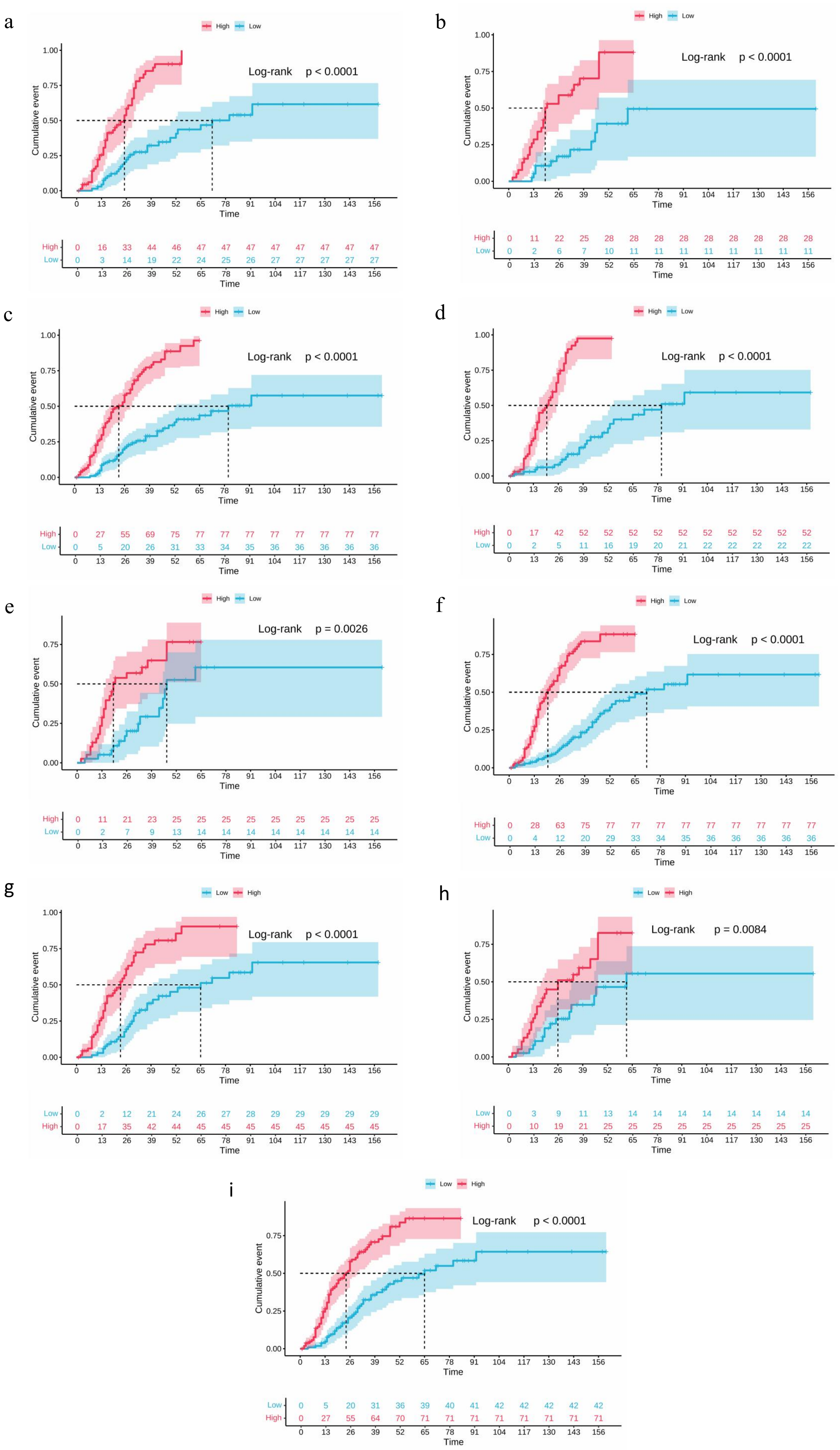

Supplementary Figure 4: (a) KM curves for patients grouped by the COX model in the training cohort; (b) KM

curves for patients grouped by the COX model in the testing cohort; (c) KM curves for patients grouped by the COX model in the ALL cohort; (d) KM curves for patients grouped by the XGBoost model in the training cohort; (e) KM curves for patients grouped by the XGBoost model in the testing cohort; (f) KM curves for patients grouped by the XGBoost model in the ALL cohort; (g) KM curves for patients grouped by the SurvSVM model in the training cohort; (h) KM curves for patients grouped by the SurvSVM model in the testing cohort; (i) KM curves for patients grouped by the SurvSVM model in the ALL cohort.
